# Recurrent acute myocarditis: An under-recognized clinical entity associated with the later diagnosis of a genetic arrhythmogenic cardiomyopathy

**DOI:** 10.3389/fcvm.2022.998883

**Published:** 2022-10-28

**Authors:** Pierre Ollitrault, Mayane Al Khoury, Yann Troadec, Yoann Calcagno, Laure Champ-Rigot, Virginie Ferchaud, Arnaud Pellissier, Damien Legallois, Paul Milliez, Fabien Labombarda

**Affiliations:** ^1^Department of Cardiology, Caen University Hospital, Caen-Normandy University, Caen, France; ^2^Department of Genetics, Caen University Hospital, Caen-Normandy University, Caen, France

**Keywords:** recurrent acute myocarditis, arrhythmogenic cardiomyopathy, myocardial inflammation, ventricular arrhythmia, sudden cardiac death

## Abstract

**Background:**

Myocardial inflammation has been consistently associated with genetic arrhythmogenic cardiomyopathy (ACM) and it has been hypothesized that episodes mimicking acute myocarditis (AM) could represent early inflammatory phases of the disease.

**Objective:**

We evaluated the temporal association between recurrent acute myocarditis (RAM) episodes and the later diagnosis of a genetic ACM.

**Materials and methods:**

Between January 2012 and December 2021, patients with RAM and no previous cardiomyopathy were included (Recurrent Acute Myocarditis Registry, NCT04589156). A follow-up visit including clinical evaluation, resting and stress electrocardiogram, cardiac magnetic resonance imaging, and genetic testing was carried out. Endpoints of the study was the incidence of both ACM diagnosis criteria and ACM genetic mutation at the end of follow-up.

**Results:**

Twenty-one patients with RAM were included and follow-up was completed in 19/21 patients (90%). At the end of follow-up, 3.3 ± 2.9 years after the last AM episode, 14/21 (67%) patients with an ACM phenotype (biventricular: 10/14, 71%; left ventricular: 4/14, 29%) underwent genetic testing. A pathogenic or likely pathogenic mutation was found in 8/14 patients (57%), 5/8 in the Desmoplakin gene, 2/8 in the Plakophillin-2 gene, and 1/8 in the Titin gene. Family history of cardiomyopathy or early sudden cardiac death had a positive predictive value of 88% for the presence of an underlying genetic mutation in patients with RAM.

**Conclusion:**

RAM is a rare entity associated with the latter diagnosis of an ACM genetic mutation in more than a third of the cases. In those patients, RAM episodes represent early inflammatory phases of the disease. Including RAM episodes in ACM diagnosis criteria might allow early diagnosis and potential therapeutic interventions.

## Introduction

Arrhythmogenic cardiomyopathy (ACM) is a genetically-determined or acquired (*e.g.*, cardiac sarcoidosis, Chagas disease, myocarditis etc.) arrhythmogenic disorder of the myocardium. Genetic ACM is the consequence of pathogenic variants in genes encoding for desmosomal and cytoskeleton proteins. Originally described as a right ventricular disease (arrhythmogenic right ventricular cardiomyopathy/dysplasia; ARVC), genetic ACM is increasingly recognized as a left or biventricular entity (1). Genetic ACM may manifest with various clinical presentations, with a pre-disposition to acute and chronic heart failure, atrial and ventricular arrhythmias or sudden cardiac death. Pathophysiology of genetic ACM at early stages remain widely unknown and, since the first pathological descriptions, myocardial inflammation has been consistently associated with ACM pathophysiology. However, the exact nature of their association remains complex and elusive (2–9). Some patients might experience recurrent episodes of chest pain with cardiac troponin elevation and signal abnormalities on cardiac magnetic resonance imaging (CMRi), labeled as recurrent acute myocarditis (RAM) or myocarditis-like syndrome. However, the temporal association between RAM episodes and the later diagnosis of a genetic ACM is unknown, especially in patients without previously known cardiomyopathy (10–14).

Therefore, we aimed to study the incidence of both ACM phenotype and ACM genetic mutation in a longitudinal cohort of patients with RAM episodes without previously known cardiomyopathy.

## Materials and methods

### Population screening

Between January 2012 and December 2021, we screened consecutive patients admitted to our tertiary university center for clinically-suspected acute myocarditis (AM), defined by at least one clinical presentation and one diagnostic criterion from the European Society of Cardiology diagnostic criteria (15). We performed a longitudinal analysis, both retrospectively by collecting previous episodes of AM (by the use of medical records) and prospectively (using the referring cardiologist follow-up). The collected study data included anamnestic and clinical findings, serum myocardial necrosis markers detection (troponin I), 12-lead electrocardiograms (ECGs), two-dimensional and Doppler echocardiogram, telemetry records, 24-hours Holter ECG and CMRi. A focus was made on family history regarding heart transplantation, sudden cardiac death before the age of 35-year-old, or any unexplained cardiomyopathy (*i.e.*, not explained by ischemic, valvular, or hypertensive heart disease). No endomyocardial biopsy was routinely performed in our center.

### Inclusion criteria

Patients were included in the present study in case of RAM, defined by more than one episode of AM requiring hospital admission. Index AM was defined as the first episode of AM. Patients were excluded in case of pre-existing cardiomyopathy or angiographically detectable coronary artery stenosis (≥50%). A follow-up program was carried out in patients with RAM, the last follow-up visit being at least 6 months after last RAM episode. Last follow-up visit included clinical examination, resting 12-lead ECG, 24-hour Holter monitoring, exercise test and CMRi. CMRi was performed on a 1.5-Tesla scanner (Magnetom Avanto, Siemens Medical Solutions, France). All patients underwent a dedicated study protocol for myocarditis, including balanced steady-state free precession sequences cine images for morpho-functional evaluation, T1-weighted turbo spin-echo sequences for detection of myocardial fat infiltration, triple inversion recovery sequences for detection of myocardial oedema and early and late gadolinium enhancement (LGE) detection, respectively 3 min and 15 min after contrast agent intravenous administration.

### Study endpoints

The primary endpoint of this study was the incidence of ACM diagnosis criteria at the last follow-up visit, as defined by the Padua criteria for right, left, and biventricular ACM (16). The secondary endpoint of this study was the incidence of genetic mutation in the population of patients with RAM and at least one ACM criteria at the last follow-up visit.

### Genetic testing

In patients with at least one diagnosis criteria of ACM at the end of follow-up, a genetic testing was performed using Next Generation Sequencing (NGS) method and analyzed with Genodiag. Sanger method was then used to validate the variant by NGS. The panel of genes analyzed was: ABCC9, ACAD9, ACTC1, ACTN2, ALPK3, ANKRD1, BAG3, CALR3, CAV3, CRYAB, CSRP3, CTNNA3, DES, DSC2, DSG2, DSP, DTNA, EMD, EYA4, FBN1, FHL1, FLNC, GAA, GATA4, GLA, HCN4, HEY2, JPH2, KRAS, LAMA4, LAMP2, LDB3 (ZASP), LMNA, MYBPC3, MYH6, MYH7, MYL2, MYL3, MYLK2, MYOM1, MYOZ2, MYPN, NEBL, NEXN, NKX2-5, PDLIM3, PKP2, PLN, PRDM16, PRKAG2, PTPN11, RAF1, RBM20, RYR2, SCN5A, SDHA, SGCD, SLC25A4, SOS1, SYNPO2, TAZ, TCAP, TMEM43, TMPO, TNNC1, TNNI3, TNNT2, TPM1, TTN, TTR, VCL. Pathogenicity of variants was classified according to current guidelines (grade IV being likely pathogenic and grade V being pathogenic).

### Statistical analysis

Data are presented as mean ± standard deviation or median (quartile 1–quartile 3) as appropriate, and categorical variables are given as number of patients with the attribute (percentage). For continuous variables, Student’s *t*-test or a Mann-Whitney *U* test, as appropriate, was performed for comparison between two groups. The Chi-square test was used for analysis of categorical variables. A *p* < 0.05 denoted statistical significance. Analyses and figures were conducted using IBM SPSS Statistics for Macintosh (Version 25.0, IBM, Chicago, Illinois).

### Ethics

The study protocol was compliant with the STROBE statement and registered as a clinical trial (Recurrent Acute Myocarditis Registry NCT04589156). Approval for this study was obtained from the local Ethics Committee and was in accordance with the declaration of Helsinki.

## Results

### Population

Between January 2012 and December 2021, a total of 21 patients (16 male; 76%) with RAM were included. Characteristics of patients, index AM, and first RAM are detailed in Table 1. No patients had a previous personal history of cardiomyopathy or ventricular arrhythmia. Five (24%) patients had a first-degree family history (genetic cardiomyopathy or ventricular arrhythmia or sudden cardiac death) and one (4.8%) patient had a non-first-degree family history (heart transplantation in a 4th degree relative). For one patient (4.8%) father-related family history was not available. Eighteen (86%), two (9.5%), and one (4.8%) patients experienced, respectively two, three, and five RAM episodes (accounting for a total of 47 AM episodes). The median age at the time of index and first recurrence was, respectively 23 [19–41] and 29 [21–44] year-old. The median time duration between index and first recurrence was two [1–5] years. Considering index AM and first RAM (42 episodes), clinical presentation was pseudo-acute coronary syndrome (ACS) and palpitations in respectively, 40/42 (95%) and 2/42 (5%) episodes. A potential infectious trigger was present in 10/42 (24%) episodes. Acute ST segment elevation and T-wave inversion were present in respectively, 16/42 (38%) and 12/42 (29%) episodes. ECG remained normal in 11/42 (26%) episodes. Sustained ventricular arrhythmia was found in 2/42 (4.8%) episodes. LGE was found in 33/42 (79%) episodes, 32/33 (99%) involving the subepicardial layer of the left ventricular lateral wall.

**TABLE 1:** Characteristics of patients (*n* = 21) and recurrent acute myocarditis (RAM) episodes (*n* = 42).

Pt #	Family history	Index AM				First RAM				
		Age (y–0)	Clinical presentation	ECG	CMR	Age (y–0)	Clinical presentation	ECG	CMRi	Other AM
1	0	20	Pseudo-ACS, Rhinitis	Diffuse ST elevation	LGE inferolateral (subepi)	22	Pseudo-ACS	TWI V4–V6	LGE lateral (subepi)	0
2	0	43	Pseudo-ACS	TWI inferolateral	LGE inferolateral basal & median	44	Pseudo-ACS	TWI inferolateral	LGE inferolateral basal	0
3	0	11	Pseudo-ACS, GE	Diffuse ST elevation	LGE lateral (subepi)	12	Pseudo-ACS	Normal	LGE inferolateral (subepi)	0
4	0	60	Pseudo-ACS	Normal	LGE lateral apical (subepi)	61	Pseudo-ACS	Normal	LGE lateral basal (subepi)	0
5	Brother SCD Mother HCM	31	Pseudo-ACS	TWI V1–V2	LGE lateral (subepi), RV inferolateral dyskinesia	32	Pseudo-ACS	TWI VI–V2±V3	LGE inferior median, lateral basal (subepi), RV dyskinesia	0
6	0	14	Pseudo-ACS	Normal	LGE inferolateral median & apical (subepi)	14	Pseudo-ACS	Normal	LGE inferolateral basal (subepi)	0
7	Mother DCM and ICD	17	Pseudo-ACS	LV anterior PVCs	LGE anterolateral (subepi), LVEF 45%	19	Pseudo-ACS	LV anterior PVCs RV inferior PVCs	LGE anterolateral LVEF 28%	0
8	0	49	Pseudo-ACS	Normal	LGE anterolateral medial (subepi)	52	Pseudo-ACS	Diffuse ST elevation	No LGE Pericardial effusion	0
9	0	18	Pseudo-ACS, Amygdalitis	Diffuse ST elevation	No LGE	21	Pseudo-ACS	Normal	No LGE LVEF 45%	1
10	0	49	Pseudo-ACS	Normal	No CMR (normal coronary angiography)	54	Pseudo-ACS, Fever	Diffuse ST elevation, TWI V3–V6. DI. aVL	LGE lateral apical (subepi) LVEF 49%	0
11	0	28	Pseudo-ACS, Bronchitis	Diffuse ST elevation TWI inferior	LGE inferior basal	35	Pseudo-ACS, Vaccine	Diffuse ST elevation	LGE multifocal, RVEF 48%, LVEF 35%	0
12	0 (unknown father)	55	Palpitations	TWI inferolateral Left VT	LGE inferolateral basal (subepi), LVEF 45%	56	Pseudo-ACS	TWI inferior Left VT	LGE inferolateral basal (subepi), inferior RV	0
13	0	23	Pseudo-ACS	PVCs	LGE inferolateral basal & median (subepi), LVEF 53%, Pericadial effusion	24	Pseudo-ACS	Anterior ST elevation	LGE inferolateral basal (subepi), anterior apical (subepi)	0
14	0	20	Pseudo-ACS	Diffuse ST elevation TWI V3–V6	LGE lateral apical (subepi)	29	Pseudo-ACS	Normal	LGE lateral apical (subepi)	0
15	Son AM Mother SCD Uncle HTx	27	Pseudo-ACS	Lateral ST elevation	No CMR (normal coronary angiography)	44	Pseudo-ACS	TWI lateral	LGE anterolateral (subepi), inferior (subepi)	0
16	Sister HTx Brother SCD Sister ICD	41	Pseudo-ACS	Normal	No CMR (normal coronary angiography)	51	Pseudo-ACS	Lateral ST elevation, NSVT	LGE multifocal, LVEF 34%	0
17	0	19	Pseudo-ACS, Fever	Diffuse ST elevation	LGE inferolateral median (subepi)	31	Pseudo-ACS, Amygdalitis	Diffuse ST elevation	LGE anterolateral median & apical (subepi), LVEF 47%	0
18	0	19	Pseudo-ACS	Diffuse ST elevation PVCs	No LGE, RV hypokinesia, RV EDV 63 ml/m^2^, RVEF 26%	19	Palpitations	TWI V1–V3 Right VT	LGE lateral median (subepi), LVEF 55%, RV hypokinesia, RV EDV 75 ml/m^2^	0
19	Brother ACM/PKP2	29	Pseudo-ACS	TWI V1–V4 Right PVCs	No LGE, RV dyskinesia, RV EDV 105 ml/m^2^, RVEF 36%, LVEF 47%	29	Pseudo-ACS	TWI anterior and inferior	LGE inferolateral median (subepi), LVEF 45%, RVEF 44%, RV RDV 90 ml/m^2^	0
20	HTx (4th degree)	17	Pseudo-ACS	Normal	No LGE	18	Pseudo-ACS	Anterior ST elevation	LGE inferolateral basal (subepi)	3
21	0	22	Pseudo-ACS, Amygdalitis	Inferior ST elevation	LGE lateral (subepi), LVEF 52%	26	Pseudo-ACS, Pharyngitis	Lateral ST elevation	LGE lateral (subepi), LVEF 50%	0

ACM, arrhythmogenic cardiomyopathy; ACS, acute coronary syndrome; AF, atrial fibrillation; AM, acute myocarditis; AVB, atrioventricular block; BAG3, BCL2-associated athanogene 3; CMRi, cardiovascular magnetic resonance imaging; DCM, dilated cardiomyopathy; DSP, desmoplakin; GE, gastroenteritis; HCM, hypertrophic cardiomyopathy; HTx, heart transplantation; ICD, implantable cardioverter defibrillator; iRBBB, incomplete right bundle branch block; LGE, late gadolinium enhancement; LV, left ventricle; LVEF, left ventricular ejection fraction; M, major criterion for ACM; m, minor criterion ACM; NSVT, non-sustained ventricular tachycardia; PKP2, plakophilin 2; PVCs, premature ventricular contractions; RVEDV, right ventricular end-diastolic volume; RVEF, right ventricular ejection fraction; RVOT, Rightventricular outflow tract; SCD, sudden cardiac death; SND, Sinus nodedysfunction; ST, ST segment; Subepi, subepicardial; TTN, titin; TWI, T wave inversion; VT, ventricular tachycardia.

### Development of an arrhythmogenic cardiomyopathy phenotype at the end of follow-up

Follow-up was completed in 19/21 patients (90%), 3.3 ± 2.9 years after the last episode of AM. Clinical, resting, and stress ECG and CMRi data at the end of follow-up are detailed in Table 2. Considering the 19 patients with complete follow-up, 14/19 (74%) developed an ACM phenotype according to the Padua criteria, the remaining 5/19 (26%) being free of cardiovascular abnormalities. None of the patients developed extracardiac signs of sarcoidosis. Among the 14 patients with an ACM phenotype, left ventricular, or biventricular forms were found respectively, in 10/14 (71%) and 4/14 (29%) patients. Major criteria for left ventricular structural abnormalities were most commonly found (14/14; 100%), followed by left ventricular morphofunctional abnormalities (5/14; 36%), right ventricular repolarization abnormalities (3/14; 21%), and right ventricular morphofunctional abnormalities (3/14; 21%).

**TABLE 2:** Follow-up of patients with recurrent acute myocarditis (RAM) (*n* = 21).

Pt #	Family history	Follow-up					ACM criteria		Genetic testing?	Genetic mutation (Grade)
		Age (y–0)	Clinical presentation	Resting ECG	Exercise ECG	CMRi	Right	Left		
1	0	26	0	Normal	Normal	LGE lateral apical	0	1M 0m	Yes	0
2	0	46	Dyspnea	SND, 1st degree AVB, iRBBB	Left PVCs	LGE lateral basal	0	1M lm	Yes	0
3	0	13	0	Normal	Normal	*Not done*	0	0	No	−
4	0	67	0	Normal	Normal	Normal	0	0	No	−
5	Brother SCD Mother HCM	35	0	TWI VI–V4	Right PVCs	RV EDV 108 ml/m^2^ and RVOT dyskinesia, LGE inferior median	2M lm	1M 0m	Yes	DSP p.Glul343Asnfs* (IV)
6	0	22	0	Normal	PVCs	LGE inferolateral median, LVEF 49%	0	2M 0m	Yes	0
7	Mother DCM and ICD	24	Palpitations	Paroxysmal AF	Left PVCs	LGE inferior basal median and anterolateral median, LVEF 28%	0	2M lm	Yes	DSP c.3082_3084+13del (IV)
8	0	No follow-up data								
9	0	23	0	Normal	Normal	Normal	0	0	No	−
10	0	69	Dyspnea	Normal	Normal	LGE lateral apical	0	1M 0m	Yes	0
11	0	35	Chest pain	Normal	Normal	LGE inferolateral basal, septal median	0	1M 0m	Yes	0
12	0 (unknown father)	63	0	TWI inferior Microvoltage	PVCs	LGE inferolateral basal, LVEF 50% LGE inferior RV	1M 0m	2M 2m	Yes	DSP C.3403OT (IV) BAG3 C.155OG (III)
13	0	25	0	rS inferolateral Microvoltage	Normal	LGE lateral basal & apical and inferior median and anterior apical	0	1M lm	Yes	0
14	0	29	0	Normal	Normal	Normal	0	0	No	–
15	Son AM Mother SCD Uncle HTx	49	Palpitations	Normal	Normal	LGE lateral median, LVEF 46%	0	2M 0m	Yes	DSP c.2588T>C (IV)
16	Sister HTx Brother SCD Sister ICD	53	0	Normal	Left PVCs	LGE multifocal, LVEF 49%	0	2M 0m	Yes	DSP c.5028_5031del (IV)
17	0	36	0	Normal	Normal	Normal	0	0	No	–
18	Sister ACM/PKP2*	23	0	TWI VI–V2 NSVT	Normal	LGE lateral median RVOT hypokinesia, RVEF 30%	2M lm	1M 0m	Yes	PKP2 C.1132C>T (V)
19	Brother ACM/PKP2	32	0	TWI VI–V6	Right PVCs	LGE inferior median RV EDV 105 ml/m^2^, RVEF 44%	2M lm	1M 0m	Yes	PKP2 C.1132C>T (V)
20	HTx (4th degree)	24	0	Normal	Normal	LGE inferolateral basal & median	0	1M 0m	Yes	TTN c.14372-2A>G (IV)
21	0	No follow-up data								

For abbreviations see Table 1.

### Prevalence of genetic mutations in recurrent acute myocarditis with arrhythmogenic cardiomyopathy phenotype

According to study protocol, 14 patients with an ACM phenotype at the end of follow-up underwent genetic testing. A pathogenic (i.e., grade V) or likely pathogenic (i.e., grade IV) mutation was found in 8/14 (57%) patients. Gene mutations were most frequently found in the Desmoplakin (DSP) gene (5/8; 63%), followed by the Plakophilin-2 (PKP2) gene (2/8; 25%), and the Titin (TTN) gene (1/8;13%): DSP p.Glu1343Asnfs*, DSPc.3082_3084 + 13del, DSP C.3403C > T, DSP c.2588T > C, DSP c.5028_5031del, PKP2 c.1132C > T (two patients from same family, brother, and sister), and TTN c.14372-2A > G. In patient #12, an additional mutation of unknown significance was found in BCL2-associated athanogene-3 (BAG3) gene.

**FIGURE 1:**
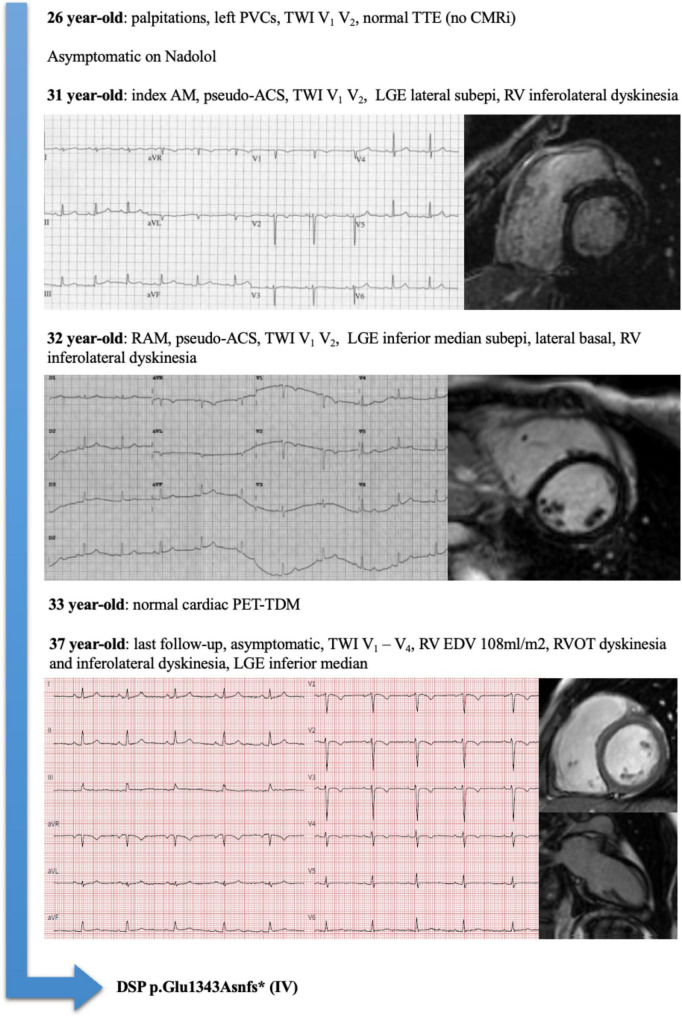
Representative example of a patient (Pt#5) with recurrent acute myocarditis (RAM), arrhythmogenic cardiomyopathy (ACM) phenotype, and Desmoplakin (DSP) mutation.

**FIGURE 2:**
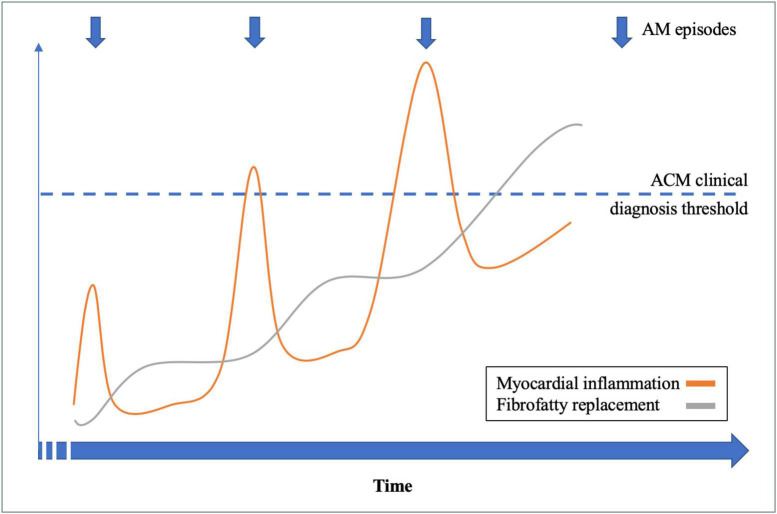
Recurrent acute myocarditis (RAM) and genetic arrhythmogenic cardiomyopathy (ACM): A proposed model.

Considering the 14 patients with RAM and ACM phenotype at the end of follow-up, family history had a sensitivity of 88% and a positive predictive value of 100% for the presence of an underlying genetic mutation. An illustrative example of a patient (number 5) with RAM, ACM phenotype, and DSP mutation is illustrated in Figure 1. A proposed model for the association between RAM and genetic ACM is illustrated in Figure 2.

## Discussion

The main findings of this study can be summarized as follow: (1) collecting data from a unique and large cohort of patients with RAM and no previous history of cardiomyopathy, we found that an ACM phenotype develops in more than 2/3rd of cases during follow-up; (2) in those patients, we found a high prevalence of previously unknown genetic mutation, illustrating the fact that that RAM represents the early phenotypic expression of a genetic ACM related to desmosomal or cytoskeleton gene mutations.

### The (under-recognized) genetic background of acute myocarditis

In the vast majority of patients, AM is an acquired cardiac disease related to a viral infection and/or an autoimmune process. It is a major cause of sudden cardiac death in young adults (17) but also a predictor of long-term adverse outcome: Grün et al. found a 19% mortality at 5 year after a biopsy proven viral myocarditis (18) and, from a large insurance database, Te et al. found a 5.4% risk of VT and a 6.5% risk of cardiovascular death at 10 years after an AM (19). RAM, defined as more than one episode of AM, has been the subject of very few case reports, and we describe here the largest cohort using a longitudinal analysis of a tertiary referral center population. The genetics of AM are complex, but recently Brown et al. identified genetic mutations in pediatric cases of acute heart failure presumed to be myocarditis, affecting TNNT2, MYBPC3, and TTN genes (20). Individuals with a genetic mutation tended to have poor outcomes, such as cardiac transplantation or cardiovascular death. Those findings were corroborated by Seidel et al. (21), which found genetic variants in DSP, TTN, and other genes in 22% of biopsy-proven pediatric myocarditis. Additionally, Piriou et al. found that in patients with AM and a family history of cardiomyopathy or sudden cardiac death, genetic testing revealed gene mutations during family screening (14). In our study, we found that more than approximately one third of patients with RAM had a previously unknown pathogenic or likely pathogenic mutation, mostly affecting the DSP gene. Recently, Ader et al. found a prevalence of genetic variants of 56% in patients with a single AM and ventricular arrhythmia or right ventricular abnormalities during follow-up (11). Altogether, those data support the fact that genetic background is a key player in AM pathogenesis, and that AM must not be considered solely as an acquired cardiovascular disease. Those data also underline the necessity of prolonged follow-up for AM patients, searching for an ACM phenotype and an underlying genetic mutation. Importantly, we found a significant proportion of patients with RAM, ACM phenotype, and without genetic mutation (6/14, 43%). Those patients might have what could be defined as “true” post-myocarditis acquired ACM, with persistent left ventricular scarring. The prognosis of such condition needs to be evaluated by a dedicated study.

### The role of inflammation in genetic arrhythmogenic cardiomyopathy

Myocardial inflammation is a common feature in genetic ACM but the exact nature of their association remains elusive (2, 3). Inflammatory infiltrates are found in approximately 80% of genetic right ventricular ACM (*i.e.*, ARVC) using post-mortem histological analysis (4). Moreover, 36% of patients with genetic right ventricular ACM have active myocardial inflammation on 18F-Fluorodeoxyglucose positron emission tomography (5). The exact mechanism of myocardial inflammation remains speculative, although evidence of myocardial auto-immunity has been found in the majority of genetic right ventricular ACM, due to auto-heart antibodies (6, 7). The proportion of patients with a clinical expression of myocardial inflammation (i.e., AM episodes) seems to be lower than the reported prevalence using non-invasive imaging or histological studies. In a cohort of 131 patients affected with genetic ACM (both right and left ventricular ACM), Lopez-Ayala et al. found a 4.5% prevalence of AM episode (10). Altogether, those data support the hypothesis of myocardial inflammation being a key pathophysiological component of genetic ACM. However, the factors favoring a clinically-overt inflammatory expression during the early phases of genetic ACM remain widely elusive. Clinical episodes of myocardial inflammation, mimicking AM, might be the consequence of both genetic and environmental factors. The prevalence of AM episode has been reported to be higher in case of DSP mutation (15%) (12), which is in line with our findings as DSP mutations were over-represented in patients with RAM and ACM phenotype. An infectious trigger might also explain an inflammatory expression in case of desmosomal gene mutation, as adenovirus or enterovirus DNA could be found in up to half of the case of right ventricular ACM using PCR on endomyocardial biopsy samples in previous studies (8, 9). Finally, Martins et al. found that, in patients with genetic ACM and previous AM episode(s), 50% of those episodes were exercise-triggered (22). This finding corroborates the fact that intense exercise increases age-related penetrance and arrhythmogenic risk in genetic ACM (23), but also the risk of adverse outcome after AM (24, 25). Finally, our work underlines the important dynamicity of myocardial inflammation in the early phase of genetic ACM, as most patients had dynamic changes in TWI and/or LGE during time (which is rather unusual in the late phase of genetic ACM). The complex interaction between genetic and non-genetic factors is a call for larger multicentric studies.

### Recurrent acute myocarditis: A new diagnosis criterion for genetic arrhythmogenic cardiomyopathy?

As discussed above, RAM and genetic ACM share common genetic and pathophysiological backgrounds, and we proposed a model linking those two entities (Figure 2). In our study, we found that the combination of RAM, ACM phenotype, and family history had a high positive predictive value for the presence of an underlying genetic mutation, confirming *a posteriori* the final diagnosis of genetic ACM (either left sided or biventricular). Therefore, implementation of AM episode(s) in the current criteria for genetic ACM diagnosis (1, 16) might allow a definite diagnosis in the early phase of the disease, when otherwise it is borderline using conventional criteria. Indeed, RAM and ACM criteria remain highly aspecific diagnostic criteria which can encountered in several other diseases than genetic ACM (sarcoidosis, Chagas disease, hypertrophic cardiomyopathy, etc.). However, our work support the fact that those aspecific anomalies, when associated together, justify genetic testing to establish a definite diagnosis of genetic ACM. Allowing an early diagnosis during the natural history of genetic ACM might offer the possibility to target myocardial inflammation, in order to improve penetrance and arrhythmogenic risk of the disease, but the therapeutic options need to be studied.

### Limitations

Our study is an observational longitudinal study, and therefore might suffer from selection bias. However, our center is the only tertiary referral center for AM, ACM, and genetic myocardial diseases for a population of 1.5 million inhabitants, which might have limited the impact of such bias. The small sample size of our cohort makes our findings exploratory. Even though we present here the largest cohort of RAM patients, those findings needs to be validated in larger multicentric studies, in order to allow multivariable statistical analysis. The fact that genetic testing was not performed in RAM patients without ACM criteria is another reason potentially leading to an underestimation of genetic mutation prevalence. However, without an arrhythmogenic phenotype, the clinical relevance of finding a mutation in a RAM patients would be limited regarding current knowledge. No systematic endomyocardial biopsy was performed in our patients, which might be considered as a limitation to label those episodes of chest, troponin elevation and CMRi abnormalities as “true” AM. However, the diagnostic yield of EBM is variable in both AM and ACM, due to patchy substrate, left ventricular, and/or subepicardial localization. In fact, histological diagnosis criteria for AM and ACM are nowadays frequently replaced by non-invasive tissular characterization by CMRi (15, 16).

## Conclusion

RAM is an under-recognized entity associated with the latter diagnosis of a genetically-determined ACM in more than a third of the cases. In those patients, RAM episodes represent early, paroxysmal, inflammatory phases of the disease. Genetic and environmental factors leading to a clinically-overt inflammatory expression of genetic ACM remain to be studied. AM episodes may justify a systematic genetic testing, especially in the presence of a suspicious family history.

## Data availability statement

The original contributions presented in this study are included in the article/supplementary material, further inquiries can be directed to the corresponding author.

## Ethics statement

The studies involving human participants were reviewed and approved by Comité Local d’Ethique sur la Recherche en Santé (CLERS). Written informed consent to participate in this study was provided by the participants’ legal guardian/next of kin. Written informed consent was obtained from the minor(s)’ legal guardian/next of kin for the publication of any potentially identifiable images or data included in this article.

## Author contributions

PO, PM, and FL elaborated the study protocol. PO, MA, and YC collected the study data and performed statistical analysis, and drafted the manuscript. All authors critically reviewed the manuscript.
